# Distribution of multidrug-resistant *Proteus mirabilis* in poultry, livestock, fish, and the related environment: One Health heed

**DOI:** 10.14202/vetworld.2025.446-454

**Published:** 2025-02-19

**Authors:** Ayesha Sarwar, Bilal Aslam, Sara Mahmood, Saima Muzammil, Abu Baker Siddique, Fatima Sarwar, Mohsin Khurshid, Muhammad Hidayat Rasool, James Sasanya, Sulaiman F. Aljasir

**Affiliations:** 1 Institute of Microbiology, Government College University, Faisalabad, Pakistan; 2 Department of Veterinary Preventive Medicine, College of Veterinary Medicine, Qassim University, Buraydah, Kingdom of Saudi Arabia; 3 Institute of Microbiology, University of Agriculture, Faisalabad, Pakistan; 4 International Atomic Energy Agency, Vienna, Austria

**Keywords:** animal-derived foods, antimicrobial resistance, multidrug-resistant *Proteus mirabilis*, One Health, virulence genes

## Abstract

**Background and Aim:**

The emergence of multidrug-resistant (MDR) *Proteus mirabilis* in food-producing animals and their associated environments is a growing public health concern. The indiscriminate use of antimicrobials in animal husbandry exacerbates resistance development, posing significant threats to food safety and sustainability. This study investigates the distribution, antibiotic resistance patterns, and virulence-associated genes (VAGs) of *P. mirabilis* isolated from poultry, livestock, fish, and their environments in Pakistan under a One Health perspective.

**Materials and Methods:**

A total of 225 samples were collected from poultry (n = 100), livestock (n = 75), and aquatic sources (n = 50) from March 2023 to September 2024. Standard microbiological methods were employed for the isolation and identification of *P. mirabilis*. Polymerase chain reaction (PCR)-based detection of antibiotic resistance genes and VAGs was performed using specific primers. Antibiotic susceptibility was assessed through the disk diffusion method following Clinical and Laboratory Standards Institute 2022 guidelines. Statistical analyses, including analysis of variance and correlation models, were applied to assess the relationships between variables.

**Results:**

*P. mirabilis* was detected in 28.44% (64/225) of the total samples, with the highest occurrence observed in poultry (38%), followed by livestock (22.67%) and aquatic sources (18%). Resistance to ampicillin (100%), chloramphenicol (82%), cefepime (75%), and ciprofloxacin (75%) was widespread. PCR analysis revealed a high occurrence of extended-spectrum beta-lactamase-producing *P. mirabilis* carrying *bla*_CTX-M_ (49%), *bla*_OXA_ (54%), and *bla*_TEM_ (25.67%) genes. In addition, VAGs such as *zapA* (39.53%), *ucaA* (34.88%), and *hpmA* (32.55%) were frequently identified. The presence of MDR *P. mirabilis* in fish and related environments (18%) is alarming, highlighting potential zoonotic and foodborne transmission risks.

**Conclusion:**

The study underscores the widespread distribution of MDR *P. mirabilis* in animal-based food sources, raising significant concerns regarding food safety and antimicrobial resistance. The findings reinforce the need for stringent monitoring and regulatory policies to mitigate MDR bacterial dissemination across the food supply chain. Future research should employ metagenomic approaches for comprehensive surveillance and risk assessment.

## INTRODUCTION

Antimicrobial resistance (AMR) is a silent epidemic highlighting a critical global health issue. Studies have revealed a direct correlation between AMR and antibiotic residues, which has resulted in its transmission in various food supply chains, subsequently showing potential danger to consumer health [1]. Globally, due to the rapid increase in the human population, ensuring safe, healthy, and clean food is becoming impossible. On a commercial basis and regarding product relatedness, it is necessary to monitor the quality of livestock, poultry, and fish meat. All handling steps, including pre-and post-slaughtering and improper utensils handling, especially during the cutting of carcasses and their by-products, required proper quality checks [2], which may play a crucial role in contamination with multidrug-resistant (MDR) bacteria [3, 4].

*Proteus mirabilis* is a Gram-negative facultative, zoonotic pathogen, the second most prevalent member of *Enterobacterales* [5], and it serves not only as a commensal microbe in human gut microflora but also in environmental samples (wastewater and soil, etc.) [6]. Furthermore, it is the most common cause of urinary tract infections (UTIs) in both hospital and community-acquired environments [7]. Region-wise variation may occur regarding the spread of *P. mirabilis* [8]. In addition, it acts as a reservoir in the intestinal tract of broiler chickens, which may be capable of transmitting during slaughtering and processing [9, 10].

Scarce data regarding the presence of *P. mirabilis* in livestock and fish are presented. Due to *Salmonella* and *Escherichia coli*, the most often studied pathogens, this bacterium is neglected. However, few studies have reported its existence in poultry, chicken, meat, and aquatic products like fish [11–14]. This strengthens the notion of cross-contamination during slaughtering and the zoonotic health significance of these strains from various meat products, which directly affect consumer health.

The present study aimed to isolate *P*. *mirabilis*, particularly from various livestock, fish, and associated environment specimens collected, followed by the detection ofantibiotic resistance genes (ARGs)and virulence-associated genes (VAGs) to determine the possible health risk of food contamination by MDR pathogens.

## MATERIALS AND METHODS

### Ethical approval

Prior consent from the Ethical Review Committee (ERC) of Government College University Faisalabad (GCUF), Pakistan, was approved (Ref No. GCUF/ERC/141, Date: February 03, 2023). All samples were obtained with preliminary approval from the stakeholders. All procedures were performed in the “OH-AMR lab; One Health AMR laboratory” GCUF.

### Study period and location

The study was conducted from March 2023 to September 2024, involving various sources of poultry, livestock, fish, and their environments in Pakistan. The samples were processed at One Health AMR Lab, Institute of Microbiology, Government College University Faisalabad Pakistan.

### Sample processing

Overall, 225 samples were collected in sterile containers from different animal-based foods, comprising a poultry group (n = 100), further divided into chicken meat, chicken carcass, cloacal swabs, and droppings; n = 25 each. The second livestock group (n = 75) was subdivided into beef, mutton, and manure/sludge, n = 25 each. Finally, the aquatic group (n = 50) was divided into raw fish and fish market waste, n = 25 each (Table 1). For each meat sample, 10 g of each sample was homogenized with 90 mL of phosphate buffered saline, incubated, and transferred in ice bags to the laboratory for further processing.

**Table 1:** Occurrence and distribution of *P. mirabilis* in selected food categories.

Specimen category	Sources	Collected samples	Positive sample	Distribution of *P. mirabilis* positive samples (%)	Distribution of *P. mirabilis* in the selected categories (%)	p-value
Poultry	Chicken meat	25	7	28	38/100 (38)	0.05*
	Chicken carcass	25	11	44		
	Cloacal/anal swabs	25	12	48		
	Droppings	25	8	32		
Livestock products	Beef	25	5	20	17/75 (22.67)	
	Mutton	25	4	16		
	Manure sludge	25	8	32		
Aquatic products	Raw fish	25	4	16	9/50 (18)	
	Fish market waste	25	5	20		
Total		225	64		64/225 (28.44)	

* Significant results among *P. mirabilis* isolates from food samples of different animal origin, *P. mirabilis*=*Proteus mirabilis*

### Isolation and identification of *P. mirabilis*

Each sample was first incubated in tryptose soya broth at 37°C overnight; further samples were inoculated on xylose-lysine-deoxycholate agar and MacConkey agar (Oxoid^®^, UK), followed by 24 incubations at 37°C. Furthermore, cultural and morphological characterization followed by biochemical identification was performed using API 20E kits (Biomeurex, Craponne, France) as per the manufacturer’s instructions.

### VAGs-based detection

The detection of different VAGs for *P. mirabilis*, including *ucaA*, *atfA*, *mrpA, hpmA (fimbriae) zapA*, *ptA* (proteases), *ireA* (siderophore receptor), and *FliL* (flagella), was conducted through polymerase chain reaction (PCR) using specific primers [15–18] (Table 2) [15–23]. Isolates were recorded as confirmed when a minimum of six *P. mirabilis*-specific VAGs were observed.

**Table 2:** Detail of primers (ARGs, VAGs) used in the study (*P. mirabilis*).

S. No.	Antibiotics/VAGs	Target	Distribution (%)	Sequence	Annealing temperature (°C)	Amplicon Size	References
				ARGs			
1	β-lactams	*bla*_CTX-M-1_	25	F: TCAAGCCTGCCGATCTGGT	55	561	[19]
				R: TGATTCTCGCCGCTGAAG			
		*bla*_TEM_	14.06	F: GGGGATGAGTATTCAACATTTCC	55	861	
				R: GGGCAGTTACCAATGCTTAATCA			
		*bla*_OXA_	14.06	F: TTGAAGGAACTGAAGGTTGT	55	651	
				R: CCAAGTTTCCTGTAAGTGCG			
		*bla*_CMY_	9.37	F: TGGCCGTTGCCGTTATCTAC	63	868	
				R: CGTTAACGGCACGATGAC			
2	Carbapenems	*bla*_NDM-1_	1.56	F: AACGCATTGGCATAAGTCGC	58	178	
				R: AACGCATTGGCATAAGTCGC			
3	Quinolones	*qnrD*	17.18	F: CGAGATCAATTTACGGGGAATA	65	572	[19]
				R: AACAAGCTGAAGCGCCTG			
		*qnrB*	3.13	F: GATCGTGAAAGCCAGAAAGG	65	476	
				R: ATGAGCAACGATGCCTGGTA			
		*qnrA*	6.25	F: CCAGGATTTGAGTGACAGC	65	592	
				R: TCCCAAGGGTTCCAGCA			
4	Sulfonamides	*sul*1	3.13	F: GTGACGGTGTTCCGCA, TTCT	72	779	[20]
				R: GGTAACATTTTCGGTTCCTG			
		*sul*2	21.87	F: CATCATTTTCGGCATCGTC R: TCTTGCGGTTTCTTTCAGC	72	793	
5	Tetracycline	*tetA*	7.81	F: : GCTACATCCTGCTTGCCTTC	63	210	[21]
				R: : CATAGATCGCCGTGAAGAGG			
		*tetB*	-	F: TTGGTTAGGGGCAAGTTTTG	63	406	
				R: GTAATGGGCCAATAACACCG			
6	Aminoglycoside	*Acc*	18.75	F: ATGACCTTGCGATGCTCTATGA R: CGAATGCCTGGCGTGTTT	54	486	[22]
7	Colistin	*mcr*-I	-	F: CGGTCAGTCCGTTTGTTC R: CTTGGTCGGTCTGTAGGG	51	309	[23]
				VAGs			
1	Fimbrae	*ucaA*	34.88	F: GCTTTTACATCCCCAGCGGT	60	476	[15]
				R: GCTGCATTTGCTGGCTCATC			
		*atfA*	20.93	F: CATAATTTCTAGACCTGCCCTAGCA R: CTGCTTGGATCCGTAATTTTTAACG	50	382	
		*mrpA*	27.9	F: ATTTCAGGAAACAAAAGATG	57	881	
				R: TTCTTACTGATAAGACATTG			
		*hpmA*	32.55	F: GTTGAGGGGCGTTATCAAGAGTC R: GATAACTGTTTTGCCCTTTTGTGC	55	709	
2	Siderophore	*ireA*	30.23	F: AAAGGGCGAGCGATTATGTATGG R: ATTGGCGCTATGTTTTGGTGTCA	55	387	[16]
3	Flagella	*FliL*	9.3	F: CTCTGCTCGTGGTGGTGTCG R: GCGTCGTCACCTGATGTGTC	57	770	[17]
4	Protease	*zapA*	39.53	F: TGGCGCAAATACGACTACCA R: TATCGTCTCCTTCGCCTCCA	57	323	[18]
		*ptA*	23.25	F: CCACTGCGATTATCCGCTCT R: ATCGGCAGAAGTGACAAGCA	60	686	

ARGs=Antibiotic resistance genes, VRGs=Virulence-associated genes, *P. mirabilis*=*Proteus mirabilis*

To achieve this, DNA extraction was performed using a K0722-DNA purification kit (Thermo-Scientific™, USA). Thermo-cycler:48 (T3000, Biomerta™, Germany) was set to run the reaction (specific VAG-annealing-temp) with the following conditions: initial denaturation for 5 min at 94°C, with subsequent 35 cycles of 1 min denaturation at 94°C, 55 s annealing, 1 min extension at 72°C and last 10 min extension at 72°C with following PCR reaction mix of 25 μL: isolate DNA 5 μL, primers 1 μL each (F & R), 8 μL DreamTaq (Thermo-Scientific™), and 10 μL Nuclease free water (Thermo-Scientific™). PCR amplicons were observed by 1.5% agarose (CSL-AG500; Rugby, UK) gel electrophoresis using a gel trans-illuminator (Bio-Rad, USA).

### Antibiotic susceptibility testing

Antibiotic susceptibility testing of *P. mirabilis* isolates was performed using a disk diffusion assay, as recommended by the Clinical and Laboratory Standards Institute 2022 [24]. Details of the antibiotics used in the study are presented in Table 3, whereas *E. coli* American Type Culture Collection (ATCC)™ 8739 (ATCC, USA) was kept as an internal control. In addition, the broth microdilution method was employed to quantify the minimum inhibitory concentration (MIC) of various antibiotics used in the study.

**Table 3:** Antibiotic susceptibility testing of *P. mirabilis* from various animal-based food origin.

Antibiotics	Conc.	CLSI/EUCAST/FDA resistance breakpoints	Chicken meat	Chicken carcass	Cloacal/anal swabs	Droppings	Beef	Mutton	Manure sludge	Raw fish	Fish market waste/sludge	Resistance (%)
Ampicillin	10 µg	≥32	128	256	512	128	256	512	256	128	128	100
Cefepime	30 µg	≥16	128	128	128	128	128	256	128	32	64	75
Ciprofloxacin	5 µg	≥4	32	64	64	32	32	64	128	32	64	75
Levofloxacin	5 µg	≥4	64	64	64	64	64	32	64	64	32	45
Chloramphenicol	30 µg	≥32	64	128	64	128	64	128	64	128	64	82
Trimethoprim	5 µg	≥16	32	32	32	64	128	64	32	64	32	55
Colistin	10 µg	≥8	0	0	0	64	0	0	0	0	0	40

*P. mirabilis*=*Proteus mirabilis*, FDA=Food and Drug Administration, CLSI=Clinical and Laboratory Standards Institute

### Molecular characterization of ARGs

Following the phenotypic susceptibility testing, isolates were subjected to genotypic detection of resistance determinants by amplifying ARGs by PCR using specific primers including extended-spectrum beta-lactamases (ESBLs): *bla*_CTX-M_, *bla*_SHV_, *bla*_TEM_, *bla*_OXA_, *bla*_CMY_, metallo-β-lactamases (MBLs): (*bla*_NDM_, *bla*_KPC_, *bla*_OXA_, *bla*_VMP_, *bla*_IMP_, *Qnrs*: *gyrA*, *gyrB*, *qnrD*, *qnrB*, *qnrA*, *sul*1,2, *tetA*, *B*, *mcr*-1, and *acc* (Table 2). For the PCR reaction mix, the 5 μL of extracted DNA was mixed in a total of 25 μL mixture with Green DreamTaq mix 10 μL (ThermoFisher Scientific, USA), primers 1 μL each, and 8 μL of nuclease-free water. Finally, 1.5% agarose gel was prepared, and the PCR products were observed under a trans-illuminator (Bio-Rad).

### Statistical analysis

The data were imported into Excel (Microsoft Office 365, Microsoft Office, Washington, USA) spreadsheets for statistical analysis. Categorical data, including the distribution of Proteus mirabilis isolates among different sample categories, were analyzed using the Chi-square (χ²) test to determine significant associations between sample types and bacterial distribution. Antibiotic susceptibility results are expressed as percent resistance (%), and differences among sample groups were evaluated using one-way analysis of variance (ANOVA) followed by Tukey’s *post hoc* test for multiple comparisons. A p-value < 0.05 was considered statistically significant. For further risk assessment, binary logistic regression analysis was performed to estimate the occurrence of MDR *P. mirabilis* in different animal-based food sources. In addition, a linear regression model was applied to predict the potential association between ARGs and VAGs in bacterial isolates, considering 95% confidence intervals (CIs). Graphical representations, including heatmaps, dot plots, and dendrograms, were constructed to illustrate the clustering of resistance genes, virulence factors, and bacterial prevalence across different sample sources. All statistical analyses adhered to standard epidemiological and microbiological research practices, ensuring reproducibility and reliability of the results.

## RESULTS

### Distribution of *P. mirabilis* in animal-derived foods

**Figure 1:**
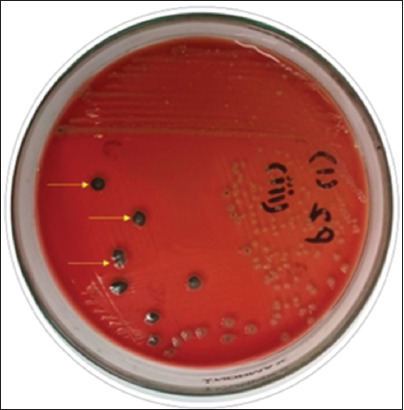
Typical black-head colonies of *Proteus mirabilis on* xylose-lysine-deoxycholate agar.

A total of 64/225 (28.44%) of the food samples tested positive for *P. mirabilis* (Figure 1) and were divided into several groups. Among these, the distribution of *P. mirabilis* was highest in poultry (38%), followed by livestock (22.67%). Furthermore, 18% of *P*. *mirabilis* were observed in aquatic products. The category-wise distribution of *P. mirabilis* is presented in Table 1.

### VAG detection

**Figure 2:**
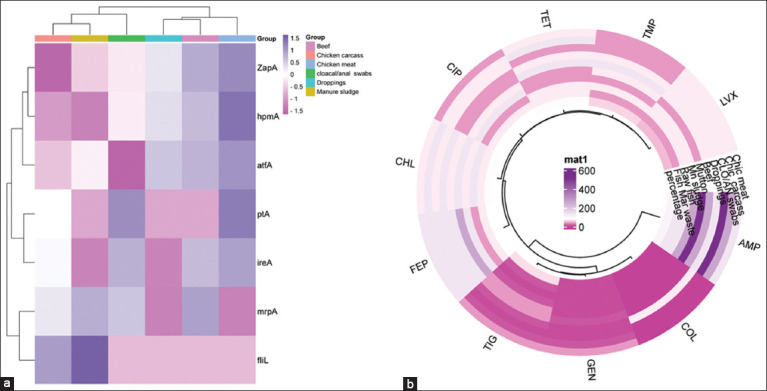
(a) Cluster heat map (double dendrogram) showing the distribution of virulence genes associated with adhesion, mobility, siderophore and protease production, iron uptake, etc., in *Proteus mirabilis* isolates from various food groups, mainly poultry (chicken meat, chicken carcass, cloacal/anal swabs, and droppings), livestock products (beef, mutton, and manure sludge), and from aquatic products (raw fish and fish market waste) shown as low, intermediate, and high on the right side. (b) The circular cluster heat map shows the resistance profile of standard antibiotics against *Proteus mirabilis* in various animal-based foods. The resistance patterns of *Proteus mirabilis* isolates are represented by color and cluster, showing overall resistance percentages against antibiotics (Ampicillin, chloramphenicol, cefepime, colistin, levofloxacin, ciprofloxacin, trimethoprim, tetracycline, tigecycline, and gentamycin).

Overall, 43 (34.4%) isolates were identified using VAGs. In poultry, 12 (48%) cloacal/anal swabs were detected with eight VRGs, followed by 11 (44%) chicken carcasses and 7 (28%) chicken meat, each carrying at least six VAGs. In addition, in livestock products, 5 (20%) beef isolates and 8 (32%) samples of manure sludge contained six VAGs. Furthermore, among all the isolates, the highest value was observed in *zapA* 17 (39.53%), followed by *ucaA* 15 (34.88%), *hpmA* 14 (32.55%), *ireA* 13 (30.23%), *mrpA* 12 (27.9%), and *ptA* 10 (23.25%), while VAGs occurrence was 9 (20.93%) in *atfA*. Finally, *FIiL* occurrence was 4 (9.3%) (Figure 2a).

### Resistance patterns of the isolates

Resistance profiling of the isolates recorded as ampicillin (100%), followed by chloramphenicol (82%), cefepime and ciprofloxacin (75%), tetracycline (70%), tigecycline (59%), trimethoprim (55%), levofloxacin (45%), and colistin (40%). The least resistance was observed in the case of gentamycin (4%) (Figure 2b and Table 3).

### Detection of ARGs

**Figure 3:**
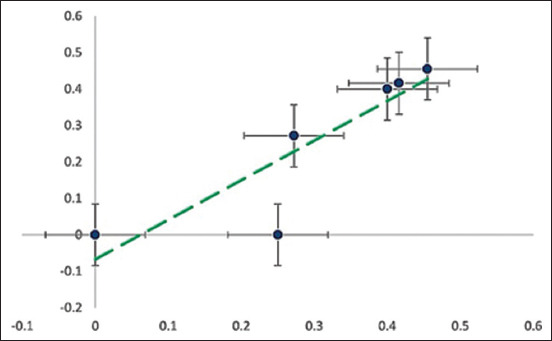
Linear regression presenting the virulence-associated gene correlation, along with error bars and adjacent positions showing the relationship strength.

**Figure 4:**
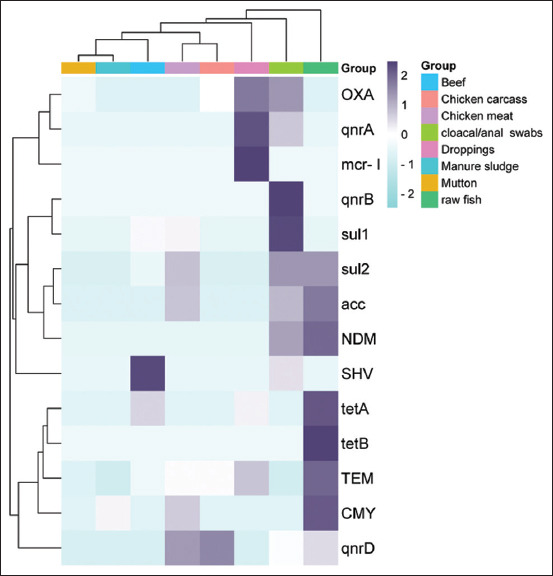
Cluster heat map (double dendrogram) showing the placement of three major categories of isolates from various animal-based foods shown in colored boxes on the right side, along with antibiotic resistance genes including extended-spectrum beta-lactamases, metallo-β-lactamases (NDM), *qnrs, sul, tet*, *mcr* (1,11), and *acc* shown in colored boxes on the right size. These genes were grouped as low, intermediate, and high frequency.

**Figure 5:**
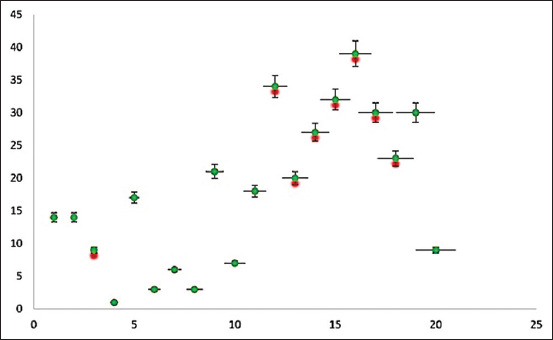
Dot plot showing the correlation between selected antibiotic resistance genes and virulence-associated genes, along with error bars and intensity of the red color under the dots showing the relationship strength.

Predominantly, the most prevalent group detected was ESBLs, and the following resistance patterns were noted in poultry, i.e., *bla*_CTX-M_ (49%)*, bl*a_TEM_ (25.67%), *bl*a_OXA_ (54%), and *bla*_CMY_ (29.95%). The only MBL detected was *bla*_NDM_ in raw fish (11.23%) and cloacal swabs (8%). *qnrs* genes were observed only in the case of poultry samples where *qnrD* was present in 50% of isolates, followed by *qnrA* (25%) and *qnrB* only (17%), while raw fish carry *qnrD* (27.53%) (Figure 3). Likewise, the detection rates of *sul*1 were 85%, and *sul*2 was 68% in cloacal swabs, while *sul*2 was 67.25% in raw fish. In addition, tetracycline resistance gene, i.e., *tet*A was observed in 67.75% and *ac*c (74.16%) of raw fish, while *acc* was observed in chicken meat (45.35%) and cloacal swabs (48.75%) (Figure 4). In addition, different ARGs were correlated with selected VAGs (Figure 5), whereas a strong correlation was observed among VAGs (Figure 3).

## DISCUSSION

Globally, foodborne pathogens represent a considerable public health concern and pose a threat to human health. Reports by the World Health Organization have declared that 42,000 deaths and 60 million morbidities occur due to foodborne microbes annually [1]. The urge to fulfill the expanded need for meat protein is directly connected to the increasing demand for chicken and other meat throughout the world. According to an approximate, estimated drastic increase expected in chicken and meat production, up to 725% in South Asian countries such as Pakistan and India, by 2030. Unhygienic and inadequate food safety practices in food supply chains may be the main cause of foodborne infections. In this study, we set up data that described the distribution of MDR *P. mirabilis*, which, after *E. coli*, is the most prevalent microbe isolated from poultry, livestock, and raw fish.

Of all the studied sources, the highest distribution of resistant *P. mirabilis* was found in chicken, with >50% of isolates showing resistance to antimicrobials related to the classes of penicillin, cephalosporins, and sulfonamides. The high prevalence of antimicrobial-resistant *P. mirabilis* in chicken is a direct consequence of the use of antimicrobials in poultry [25, 26]. In addition, some of the veterinary medicines such as enrofloxacin, florfenicol, and ceftiofur are related to the same antimicrobial group used in the treatment of human infections, revealing cross-resistance and endorsed the one health concept to rectify and slow down the influence of resistance against different antimicrobials.

Among the various animal-based food sources analyzed in this study, the poultry sector was the main cause of the expansion of MDR *P. mirabilis*, with a 38% resistance rate. In addition, livestock showed a lower resistance (17%) pattern, followed by aquatic products (9%). The most probable causes are expansive nurturing and insufficient consumption of beef/mutton and raw fish [27, 28].

Furthermore, among poultry samples, the highest distribution of MDR *P. mirabilis* was observed in cloacal/anal swabs (48%), which is in line with a previous study by Sajjan and Srinivasa [29]. For chicken carcasses, 44% resistance pattern was observed in *P. mirabilis*, which is similar to reports from other studies in North Brazil [18] and Pakistan [30]. Another study was carried out regarding the presence of MDR *P. mirabilis*, i.e., 39% in poultry dropping, which is approximately similar, i.e., 32% with our records [31]. In addition, another study by Ma *et al*. [32] indicated that 19% of *P. mirabilis* was present in chicken meat, which coincides with our study finding of 28%.

The current study showed 20% MDR *P. mirabilis* in beef samples, which agrees with but slightly less than the findings by Sanches *et al*. [14], i.e., 27.80%, whereas Chinnam *et al*. [33] exhibited the existence of a very lower percentage of MDR *P. mirabilis* in mutton (9.18%), which is identical to our results showing (16%), the least among livestock products, whereas animal manure showed 32%, which coincided with a finding by Sardari *et al*. [34], i.e., 16.2%. Finally, regarding aquatic products, the present study showed a 16% and 20% prevalence of MDR *P. mirabilis*, which coincides with a previous study by Ma *et al*. [32].

This study revealed the highest rate of resistance of ESBL-producing isolates to different antibiotics (Figure 2b). The same findings in chicken carcasses and poultry farms were reported by Li *et al*. [35]. Similarly, *bla*_CTX-M_- and *bla*_TEM_-harboring variants also showed higher resistance to different antibiotics compared with the non-harboring strains. The current findings require more attention because they indicate that antimicrobial use other than beta-lactams in animal production may increase the emergence of ESBL-harboring strains. Likewise, a study described that MDR *P. mirabilis* strains producing *bla*_CTX-M_, *bla*_TEM_, and *bla*_CMY_ recovered from chickens, meat, and aquatic products showed pattern resistance against antibiotics other than beta-lactam drugs [32].

The major contributors regarding virulence genes in this study were related to fimbriae encoding genes (Table 2) such as *ucaA*, *atfA*, *mrpA*, and *hpmA*, which co-relate with a study by Pellegrino *et al*. [36] in humans with UTI. Another study by Barbour *et al*. [37] showed the presence of the *mrp*A gene in all MDR *P. mirabilis* isolates recovered from chickens and humans and identified significant homology, suggesting the possible zoonotic dynamics of this pathogen. Although the pathogen may not cause UTI due to temperature variation, *atfA* expression may be helpful for the environmental stability of *P*. *mirabilis* [38].

Overall, an array of VAGs was observed in the isolates of the study, including proteases and hemolysin siderophore receptors, suggesting the diverse nature of *P. mirabilis*, particularly among poultry. In contrast to hemolysin, none of the isolates displayed the presence of *hlyA*. These findings are similar to previously reported data showing *hpmA* as a dominant VAG [17]. Furthermore, sporadic research is conducted on the virulence factors associated with food infection by MDR *P. mirabilis*. However, Sanches *et al*. [18] revealed VAGs in MDR *P. mirabilis* recovered from animal-based foods.

Taking these findings together, the distribution of MDR *P. mirabilis* among food sources such as chicken, livestock, and fish is worrisome and requires special attention from stakeholders. Food contamination with resistant bacterial pathogens is a serious public health concern because the associated environment also plays a part in the dissemination of resistant bacteria and ARGs, as evidenced by the findings of the present study, which confirms the one health significance of this pathogen. In the future, a comprehensive metagenomic surveillance study may be conducted by employing a holistic One Health approach, which may be helpful in estimating the precise burden and crafting a control strategy for this health threat.

## CONCLUSION

This study provides crucial insights into the occurrence, AMR patterns, and VAGs of *P. mirabilis* isolated from poultry, livestock, fish, and their associated environments in Pakistan. The findings underscore the alarming distribution of MDR *P. mirabilis*, with an overall occurrence of 28.44% across all sample categories. The highest contamination was recorded in poultry (38%), followed by livestock (22.67%) and aquatic sources (18%). Antibiotic susceptibility profiling revealed a high resistance rate to ampicillin (100%), chloramphenicol (82%), and third-generation cephalosporins (75%), with ESBL-producing *P. mirabilis* carrying *bla*_CTX-M_ (49%), *bla*_OXA_ (54%), and *bla*_TEM_ (25.67%) genes. Moreover, the detection of virulence markers such as *zapA* (39.53%) and *ucaA* (34.88%) suggests an enhanced pathogenic potential of these isolates, increasing the risk of zoonotic transmission.

The study offers valuable contributions by employing a comprehensive One Health approach, integrating molecular characterization of ARGs and virulence factors, and utilizing advanced statistical models to analyze resistance patterns. These findings contribute to global AMR surveillance and provide evidence-based recommendations for policymakers and public health authorities. However, the study is geographically limited to Pakistan, and findings may not be generalizable to other regions with different antimicrobial usage patterns. The sample size, while providing significant insights, could be expanded in future studies to strengthen epidemiological conclusions. The study primarily relied on phenotypic resistance and PCR-based gene detection, whereas whole-genome sequencing (WGS) could provide deeper insights into genetic variations and resistance mechanisms. In addition, the role of environmental factors in AMR transmission requires further exploration.

Future research should expand the study to broader geographic regions for nationwide and cross-border AMR surveillance in food supply chains. The application of metagenomic and WGS approaches would allow for a deeper understanding of the complete resistome and mobilome of *P. mirabilis* strains. Evaluating alternative antimicrobial interventions, such as bacteriophage therapy or probiotics, could help mitigate the spread of resistant strains. Longitudinal studies monitoring resistance trends and assessing the impact of antimicrobial stewardship programs in food production sectors would be highly beneficial. Policy interventions should focus on stricter regulations regarding antimicrobial usage in veterinary and food industries to control the spread of MDR bacterial strains.

The study underscores the urgent need for integrated One Health strategies to manage the dissemination of MDR *P. mirabilis* in food-producing animals and associated environments. The increasing presence of resistant strains in animal-based food sources highlights significant public health risks, emphasizing the necessity of global AMR surveillance and evidence-based policy interventions. Future efforts should prioritize multisectoral collaboration to combat AMR and ensure food safety for sustainable public health protection.

## AUTHORS’ CONTRIBUTIONS

AS and BA: Conceptualization and methodology. SMa and SMu: Data curation and formal analysis and validation. ABS, MHR, FS, and MK: Methodology and drafted the manuscript. SA: Conceptualization, methods, and reviewed and edited the manuscript. BA, JS and MHR: Supervision and Project administration. All authors have read and approved the final manuscript.

## References

[1] Fung F, Wang H.S, Menon S (2018). Food safety in the 21^st^ century. Biomed J.

[2] Rouger A, Tresse O, Zagorec M (2017). Bacterial contaminants of poultry meat:Sources, species, and dynamics. Microorganism.

[3] Di K.N, Pham D.T, Tee T.S, Binh Q.A, Nguyen T.C (2021). Antibiotic usage and resistance in animal production in Vietnam:A review of existing literature. Trop. Anim. Health Prod.

[4] Kasimanickam V, Kasimanickam M, Kasimanickam R (2021). Antibiotics use in food animal production:Escalation of antimicrobial resistance:Where are we now in combating AMR. Med Sci. (Basel).

[5] Reu C.E, Volanski W, Prediger K.C, Picheth G, Fadel-Picheth C.M.T (2018). Epidemiology of pathogens causing urinary tract infections in an urban community in southern Brazil. Braz. J. Infect. Dis.

[6] Drzewiecka D (2016). Significance and roles of *Proteus* spp. bacteria in natural environments. Microb. Ecol.

[7] Armbruster C.E, Mobley H.L.T, Pearson M.M (2018). Pathogenesis of *Proteus mirabilis* infection. EcoSal Plus.

[8] Gajdács M, Urbán E (2019). Comparative epidemiology and resistance trends of *Protease* in urinary tract infections of inpatients and outpatients:A 10-year retrospective study. Antibiotics (Basel).

[9] Tesson V, Federighi M, Cummins E, de Oliveira Mota J, Guillou S, Boué G. A (2020). A systematic review of beef meat quantitative microbial risk assessment models. Int. J. Environ. Res. Public Health.

[10] Baéza E, Guillier L, Petracci M (202). Review:Production factors affecting poultry carcass and meat quality attributes. Animal.

[11] Yu Z, Joossens M, Van den Abeele A.M, Kerkhof P.J, Houf K (2021). Isolation, characterization and antibiotic resistance of *Proteus mirabilis* from Belgian broiler carcasses at retail and human stool. Food Microbiol.

[12] Guo S, Aung K.T, Tay M.Y.F, Seow K.L.G, Ng L.C, Schlundt J (2019). Extended-spectrum ?-lactamase-producing *Proteus mirabilis* with multidrug resistance isolated from raw chicken in Singapore:Genotypic and phenotypic analysis. J. Glob. Antimicrob. Resist.

[13] Shammah V, Mailafia S, Ameh J, Cejetan Ifeanyi C, Adebari A, Odey E, Sabo R (2023). Phenotypic and antimicrobial susceptibility studies of *Proteus mirabilis* isolates from fresh water fishes in FCT, Abuja-Nigeria. Adv. Microbiol.

[14] Sanches M.S, Rodrigues da Silva C, Silva L.C, Montini V.H, Lopes Barboza M.G, Guidone G.H.M, Dias de Oliva B.H, Nishio E.K, Faccin Galhardi, L.C. and Vespero E.C (2021). *Proteus mirabilis* from community-acquired urinary tract infections (UTI-CA) shares genetic similarity and virulence factors with isolates from chicken, beef and pork meat. Microb. Pathog.

[15] Rocha S.P.D, Elias W.P, Cianciarullo A.M, Menezes M.A, Nara J.M, Piazza R.M.F, Silva M.R.L, Moreira C.G, Pelayo J.S (2007). Aggregative adherence of uropathogenic *Proteus mirabilis* to cultured epithelial cells. FEMS Immunol. Med. Microbiol.

[16] Zunino P, Geymonat L, Allen A.G, Legnani-Fajardo C, Maskell D.J (2000). Virulence of a *Proteus mirabilis* ATF isogenic mutant is not impaired in a mouse model of ascending urinary tract infection. FEMS Immunol. Med. Microbiol.

[17] Cestari S.E, Ludovico M.S, Martins F.H, da Rocha S.P.D, Elias W.P, Pelayo J.S (2013). Molecular detection of HpmA and HlyA hemolysin of uropathogenic *Proteus mirabilis*. Curr. Microbiol.

[18] Sanches M.S, Baptista A.A.S, de Souza M, Menck-Costa M.S, Koga V.L, Kobayashi K.T.R, Rocha S.P.D (2019). Genotypic and phenotypic profiles of virulence factors and antimicrobial resistance of *Proteus mirabilis* isolated from chicken carcasses:Potential zoonotic risk. Braz. J. Microbiol.

[19] Wong M.H.Y, Hoi Y.W, Sheng C (2013). Characterization of multidrug-resistant *Proteus mirabilis* isolated from chicken carcasses. Foodborne Pathog. Dis.

[20] Jiang X, Xu Y, Li Y, Zhang K, Liu L, Wang H, Tian J, Ying H, Shi L, Yu T (2017). Characterization and horizontal transfer of qacH-associated class 1 integrons in *Escherichia coli* isolated from retail meats. Int. J. Food Microbiol.

[21] Qamar M.U, Aatika Chughtai M.I, Ejaz H, Mazhari B.B.Z, Maqbool U, Alanazi A, Alruwaili Y, Junaid K (2023). Antibiotic-resistant bacteria, antimicrobial resistance genes, and antibiotic residue in food from animal sources:One health food safety concern. Microorganisms.

[22] Edris S.N, Hamad A, Awad D.A.B, Sabeq I.I (2023). Prevalence, antibiotic resistance patterns, and biofilm formation ability of *Enterobacterales* recovered from food of animal origin in Egypt. Vet. World.

[23] Uddin M.B, Hossain S.B, Hasan M, Alam M.N, Debnath M, Begum R, Roy S, Harun-Al-Rashid A, Chowdhury M.S.R, Rahman M.M, Hossain M.M, Elahi F…, Ahmed S.S.U (2021). Multidrug antimicrobial resistance and molecular detection of MCR-1 gene in *Salmonella* species isolated from chicken. Animals (Basel).

[24] CLSI (2022). Performance Standards for Antimicrobial Susceptibility Testing.

[25] Nhung N.T, Chansiripornchai N, Carrique-Mas J.J (2017). Antimicrobial resistance in bacterial poultry pathogens:A review. Front. Vet. Sci.

[26] Hedman H.D, Vasco K.A, Zhang L (2020). A review of antimicrobial resistance in poultry farming within low-resource settings. Animals (Basel).

[27] Rabello R.F, Bonelli R.R, Penna B.A, Albuquerque J.P, Souza R.M, Cerqueira A.M.F (2020). Antimicrobial resistance in farm animals in Brazil:An update overview. Animals (Basel).

[28] Sichewo P.R, Gono R.K, Muzondiwa J (2014). Isolation and identification of pathogenic bacteria in edible fish:A case study of rural aquaculture projects feeding livestock manure to fish in Zimbabwe. Int. J. Curr. Microbiol. Appl. Sci.

[29] Sajjan S.T, Srinivasa R (2022). Antibiogram and molecular detection of beta-lactamase genes in *Proteus mirabilis* isolated from pork, chicken meat and chicken cloacal swabs. J. Pharm. Innov.

[30] Ishaq K, Ahmad A, Rafique A, Aslam R, Ali S, Shahid M. A, Sarwar N, Aslam M.A, Aslam B, Arshad M.I (2022). Occurrence and antimicrobial susceptibility of *Proteus mirabilis* from chicken carcass. Pak. Vet. J.

[31] Nahar A, Siddiquee M, Nahar S, Anwar K.S, Ialam S (2014). Multidrug resistant-*Proteus mirabilis* isolated from chicken droppings in commercial poultry farms:Bio-security concern and emerging public health threat in Bangladesh. J. Biosaf Health Educ.

[32] Ma W.Q, Han Y.Y, Zhou L, Peng W.Q, Mao L.Y, Yang X, Wang Q, Zhang T.J, Wang H.N, Lei C.W (2022). Contamination of *Proteus mirabilis* harboring various clinically important antimicrobial resistance genes in retail meat and aquatic products from food markets in China. Front. Microbiol.

[33] Chinnam B.K, Subhashini N, Srinivasa R.T, Suresh B.V, Venkata C.P, Bhavana B (2022). Detection of B-lactamase-producing *Proteus mirabilis* strains of animal origin in Andhra Pradesh, India and their genetic diversity. J. Food Protect.

[34] Sardari M, Meysam Manouchehrifar M, Hasani K, Habibzadeh N, Doghaheh H.P, Azimi T, Arzanlou M (2024). Molecular characterization and prevalence of ?-lactamase-producing *Enterobacterales* in livestock and poultry slaughterhouses wastewater in Iran. J. Water Health.

[35] Li Z, Chong P, Gerui Z, Shen Y, Zhang Y, Liu C, Liu M, Wang F (2022). Prevalence and characteristics of multidrug-resistant *Proteus mirabilis* from broiler farms in Shandong Province, China. Poult. Sci.

[36] Pellegrino R, Scavoane P, Umpiérrez A (2013). *Proteus mirabilis* uroepithelial cell adhesin (UCA) fimbria plays a role in the colonization of the urinary tract. Pathog. Dis.

[37] Barbour E.K, Hajj Z.G, Hamadeh S, Shaib H.A, Farran M.T, Araj G, Faroon O, Barbour K.E, Jirjis F, Azhar E, Kumosani T, Harakeh S (2012). Comparison of phenotypic and virulence genes characteristics in human and chicken isolates of *Proteus mirabilis*. Pathog. Glob. Health.

[38] Zunino P, Sosa V, Schlapp G, Allen A.G, Preston A, Maskell D.J (2007). Mannose-resistant Proteus-like and *P. mirabilis* fimbriae have specific and additive roles in *P. mirabilis* urinary tract infections. FEMS Immunol. Med. Microbiol.

